# Incidence of depression among community‐dwelling older adults: A systematic review

**DOI:** 10.1111/psyg.13081

**Published:** 2024-01-23

**Authors:** L.E.E. Brasileiro, Amanda Almeida Gomes Dantas, Dorothy Bezerra Linhares, Heron Alves Vale, Marc Terradas‐Monllor, Mirari Ochandorena‐Acha, Aílla Lorenna de Medeiros Paiva, Maria Yasmin Dantas de Medeiros, Javier Jerez‐Roig, Dyego Leandro Bezerra de Souza

**Affiliations:** ^1^ Postgraduate Program in Collective Health Federal University of Rio Grande do Norte (UFRN) Natal Brazil; ^2^ Multicampi School of Medical Sciences Federal University of Rio Grande do Norte (UFRN) Caicó Brazil; ^3^ Department of Collective Health, Graduate Program in Collective Health Federal University of Rio Grande do Norte Natal Brazil; ^4^ Graduate Program in Health Sciences Federal University of Rio Grande do Norte Natal Brazil; ^5^ Research group on Methodology, Methods, Models and Outcomes of Health and Social Sciences (M_3_O), Faculty of Health Sciences and Welfare, Center for Health, and Social Care Research (CESS) University of Vic‐Central University of Catalonia (UVic‐UCC) Vic Spain; ^6^ Institute for Research and Innovation in Life and Health Sciences in Central Catalonia (IRIS‐CC) Vic Spain

**Keywords:** aged, cohort studies, depression, depressive disorder, incidence, systematic review

## Abstract

We aimed to synthesise information related to the incidence of depression and depressive symptoms (DDS) in a community‐dwelling older adult population at a global level. In this systematic review, we included articles with a cohort study design that evaluated the incidence of depression or depressive symptoms in older adults aged 60 years or more in a community‐dwelling environment. Six databases were used: Web of Science, PubMed, Scopus, LILACS, SciELO, and Cochrane, and the entire selection process was independently performed by peers. We divided the included articles into subgroups according to the DDS assessment instrument: (i) Geriatric Depression Scale; (ii) Center for Epidemiologic Studies Depression Scale; (iii) miscellaneous scales; and (iv) diagnostic interviews. Each cumulative incidence value obtained per item was adjusted for a 1‐year follow‐up period, which generated an annual cumulative incidence (AcI). From 46 articles, 42 used scales to evaluate the depressive variable, with an AcI estimate of around 4.5%. The articles that assessed depression categorically observed a variation in AcI between 0.2% and 7.0%. Among all the materials included, the group that used the Geriatric Depression Scale observed the lowest and the highest AcI, 1.3% and 26.6% respectively. Most of the productions were from countries in the Asian continent (52.2%), followed by Europe (30.4%), the Americas (13%), and Oceania (4.4%). Despite the variation of AcI, we found a frequent occurrence of DDS in older adults in the community‐dwelling environment, which highlights the need for preventive actions and better‐targeted early care, especially in terms of primary health care.

## INTRODUCTION

Projections reveal that between the years 2019 and 2050, the number of older adults aged 65 years and over will double on a global level.^1^ This demographic trend signals new challenges for societies, which leverages the need for greater knowledge about entities or characteristics associated with human ageing.

Depression among older adults has peculiar characteristics, as it involves different impacts when compared to that of adults. While depression in adults is associated with disability,^2^ in older adults it is related to the loss of years lived with quality,^3^ mortality from all causes,^4^ besides being a central element that can link diseases, poor healthy behaviours, and psychological conditions.^5^

In a recent systematic review on the theme of depression in older adults, the authors sought to evaluate risk and protective factors for depression. They identified a major heterogeneity among the included studies but concluded that limitations and damages related to mobility, instrumental activities of daily living, visual acuity, as well as chronic diseases, and difficulties initiating sleep, were presented as risk factors for depression in the older adult population.^6^

In another systematic review, this time investigating depression in people 50 years old and over, the authors identified five risk factors, in brief: bereavement, sleep disturbances, disabilities, history of previous depression, and female gender.^7^ Twenty years on from this review, it appears that variables such as quality of life and loneliness may play an important role in the development of depression in older adults.^8^, ^9^

Regarding what is known about the incidence, in a systematic review, after the inclusion of 20 articles in which adults were 70 years old or more, the authors observed a variation in the incidence values according to the classification of the depressive spectrum. Thus, for ‘major depression’, the incidence ranged from 1.7% to 7.6%, while for ‘depressive symptoms’, the upper limit of total incidence ranged from 19% to 22%.^10^

The context in which older adults live can also provide specific information about the factors associated with mental illness. The depression of community‐dwelling older adults may have a worse prognosis, since in addition to the fluctuating character of depression due to possible relapses, there is a tendency to chronicity.^11^ Although there is a possibility of a better quality of life for community‐dwelling older adults when compared to those who live in institutions,^12^ this is probably an under‐treated group. Knowledge is still scarce and with controversial characteristics, despite the studies carried out.^13^ In this sense, the objective of this study was to synthesise information related to the incidence of depression and depressive symptoms in the world population, specifically among community‐dwelling older adults.

## METHODS

This study is a systematic review with initial registration in PROSPERO (International Prospective Register of Systematic Reviews) under number CRD42019121616. As a reference for this paper, we used the updated version of the PRISMA (Preferred Reporting Items for Systematic reviews and Meta‐Analyses) 2020 Statement.^14^

### Eligibility criteria

Articles in which samples were composed of older adults aged 60 years or more, who lived in a housing arrangement related to maximum independence and self‐determination of these older adults, that is, who were not institutionalised or hospitalised, were included in this review.

This review considered articles with observational and longitudinal study designs in its decision process, since they allow the assessment of cumulative incidence and incidence ratio values.

In addition to the age, setting, and study designs mentioned, there was no restriction on the language used or the publication period. To make the included studies less heterogeneous, we excluded from this systematic review articles in which the depressive variable was related to bipolar depression or depression with psychotic symptoms; studies that investigated as predictive variables chemical, molecular or blood elements; studies that evaluated groups of older adults with specific diseases or that were part of specific clinical populations; studies in which the exposure variable was associated with natural disasters or catastrophes; and finally, studies that did not perform a longitudinal assessment, even if there was a follow‐up in more than one moment of a trajectory. Furthermore, materials corresponding to grey literature and where it was not possible to access the written material, were also excluded.

### Information sources and the selection process

Six health‐related databases were searched: Web of Science, PubMed, Scopus, LILACS (Latin‐American and Caribbean Literature on Health Sciences), SciELO (Scientific Electronic Library Online), and Cochrane. We chose to associate controlled and uncontrolled terms, obtaining an equation that was individualised using the characteristics of each database. This was exposed in a publication about the protocol of this systematic review.^15^ We obtained materials that were published by December 31, 2020.

Before the beginning of the process itself, weekly or biweekly virtual meetings were established among the members of the author team in order to standardise, train, and make adjustments. The process of screening the articles to be included in this review was manual, peer‐reviewed, and independent by following these stages: reading of titles and abstracts, full texts, extraction of results, evaluation of the quality of each included article, collection of titles from the reference list of included articles. For each pair of authors, a third member participated to dispel disagreements, or if this was not possible, the pair of authors should reach a consensus decision, given the eligibility criteria. It was only possible to use this process because all 10 authors participated in this review at various stages.

Because no filters were used in the search strategy, such as types of documents, language, and types of studies, some materials from the grey literature were found, such as doctoral theses or abstracts published in conference annals. These had to be excluded due to the large number of materials that went into the full‐text selection. Conference abstract publications did not provide the necessary methodological details, or enough results for extraction, which caused their exclusion, even after contacting the authors via electronic mail, since no response was obtained. For this manuscript, articles that did not provide the incidence of depressive symptoms or depression were excluded.

### Data extraction

After concluding the study selection process, the articles to be extracted were evenly divided among eight authors, meaning that four pairs were working independently (LE and HA, AA and DB, MT and JJ, MO and DL). By the end of a certain given period, each pair was to reach a consensus. Adjustment meetings were necessary in order to standardise results. The topics extracted for each included article were: main author, year, name of the study the article was part of, study design, follow‐up time, population context, inclusion and exclusion criteria, sample size (considering losses and final sample), baseline characteristics (percentage of female gender and measures of central tendency for age), variables used to collect outcome data and cutoff point, and finally, incidence‐related information.

The values of cumulative incidence or incidence ratio were directly obtained from the articles, from the description of the results or arranged in tables. For articles that did not directly bring these values, they were obtained by calculations performed by the authors themselves.

### Bias assessment of included articles

The risk of bias in all studies included in this systematic review was assessed using the Quality Assessment Tool for Observational Cohort and Cross‐Sectional Studies (https://www.nhlbi.nih.gov/health-topics/study-quality-assessment-tools). This is a 14‐item checklist for observational studies, including cohort studies. For each item, the evaluators should identify the fulfilment in ‘yes’, ‘no’ and ‘other’ (cannot be determined; not applicable; not reported), and at the end, the number of ‘yes’ responses was quantified for each article. Six authors from the team (LE and AA; MT and JJ; MO and DL) participated in this evaluation process and in a paired and independent way reached a consensus thereafter.

For each study, we decided to quantify the points included in a final score, and for each item, we classified them as: yes, no, not reported, unclear, not applicable, and cannot be determined. This step was also performed in a paired manner and with the consensus of each pair of evaluators. It is noteworthy that this classification is important because it directs the evaluators to essential concepts related to internal validity, which includes items for potential risk of selection bias, information bias, and confounding bias. Therefore, we did not use the critical appraisal with the intent of making a summary judgement of the quality of each study.

For some items, the authors pre‐established criteria based on what the checklist provides as general guidelines. This is because even with the explanations provided by the checklist, there could be dissonance between the evaluators. In this sense, the directions for the items are as follows: 7 (as there is no accuracy in the literature, a minimum follow‐up period of 6 months was established to be considered ‘yes’), 8 (when some variables have validated instruments and cutoff points, but others do not, we considered ‘no’ – ‘yes’ would be for when all variables were validated), 14 (we chose ‘yes’ for when the adjustment was made at least for gender *and* age *and* at least another variable).

### Treatment and synthesis of included data

The method for the synthesis of the extracted data was only possible after completing the extraction and critical evaluation steps of each article, due to a large number of articles likely to be included throughout the process. Thus, we chose to analyze the cumulative incidence (cI) results in a way that decreased the heterogeneity from a theoretical point of view, according to the instrument used to obtain incidence, as well as from the evaluation of the internal validity of each article. When incidence data were not found, the value was obtained by calculating the ratio between the number of new cases by the total number of participants at risk of having depression.

From this, we analyzed the cI of each article by the ratio between the number of events over the total number of the sample at risk (older adults without depressive symptoms or depression at baseline). As there was variation in the follow‐up time between studies, the cI was adjusted to the time, dividing it by the number of years of follow‐up, which resulted in the value of an annual percentage represented here by the annual cI (AcI). These data were displayed through bar graphs. Since the objective of this review is the synthesis of data regarding the incidence of depressive symptoms/depression, the articles that did not bring the number of events (for calculation) or the value of cumulative incidence were excluded from the synthesis of frequency.

## RESULTS

**Figure 1:**
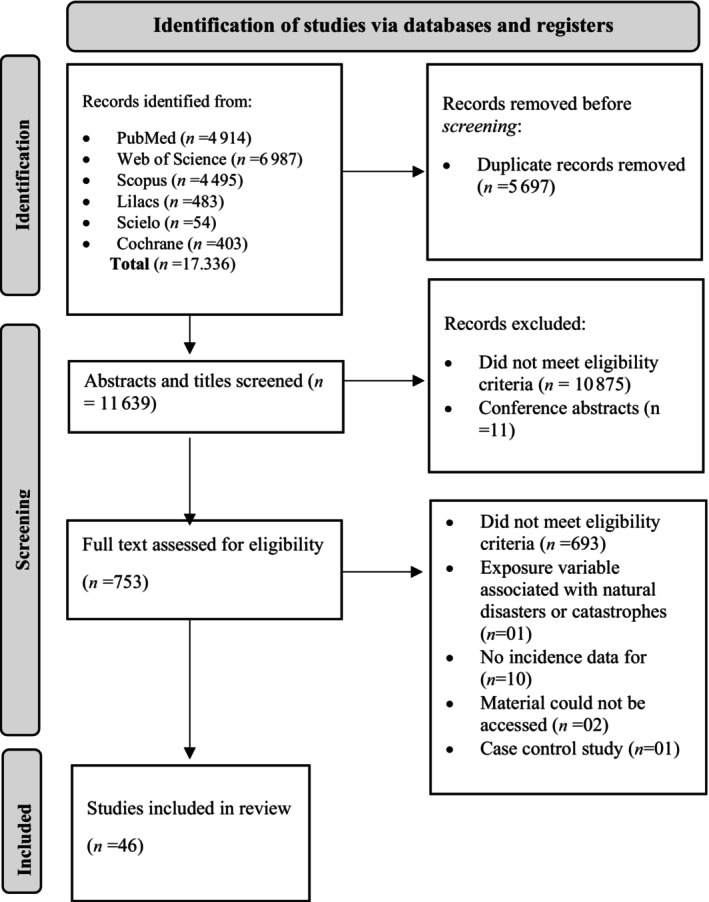
Flowchart identifying the systematic review selection process.

From the 17 336 documents found with the search strategy described for all six databases, we included 46 articles for extraction and quality assessment. It can be seen in the flowchart (Fig. 1) that after the exclusion of duplicate materials, 10 888 documents were filtered out through paired reading of titles and abstracts, of which 10 875 did not meet the eligibility criteria.

Among the 753 articles, two had to be excluded due to restricted access to Chinese platforms; the remainder was divided between six authors (MY, AP, DB, HA, LE, AA). This was followed by the exclusion of 693 articles for not being in accordance with the eligibility criteria, 10 did not respond to the objective of the article. Therefore, 46 articles were obtained.

### AcI

From the 46 articles included in this systematic review, we found that 181 719 older adults aged 60 years and over participated in the totality of the studies, among which a total of 24 948 were considered as incident cases of depression over time.

We obtained AcI data for each of the studies; however, it was not possible to use a measure of central tendency to characterise all of the included articles’ incidence results. This is due to the heterogeneity of the included studies, revealed for example by the high amplitude (25.3) among the AcI values.

In order to better understand the data obtained, the articles were grouped according to the instruments used to evaluate the depressive variable: (i) Geriatric Depression Scale‐GDS; (ii) Center for Epidemiologic Studies Depression Scale‐CES‐D; (iii) miscellaneous scales; and (iv) diagnostic instruments. In this sense, the first three subgroups present incidence results in a screening perspective when considering dimensionality, while the fourth subgroup used categorisation through diagnostic interviews. The median AcI of the first three subgroups was 4.5% (interquartile range (IQR) *q*1–*q*3: 3.5%–6.2%), whereas the median AcI (for major depression) of the fourth subgroup was 3.9% (IQR *q*1–*q*3: 0.8–6.6).

**Figure 2:**
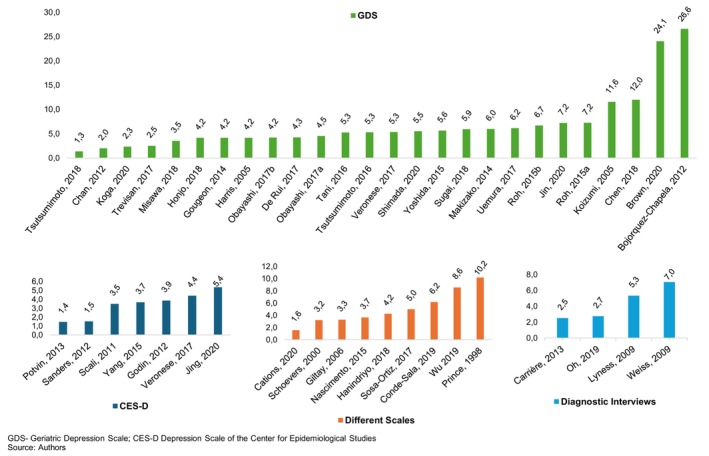
Annual cumulative incidence grouped by screening/diagnostic tools.

Based on these data, we noted that it was in the group that used the GDS that we found both the lowest and the highest AcI of depression within the included studies, 1.3% and 26.6% respectively.^16^, ^17^ The AcI values of this group also represent a greater heterogeneity among the four groups, in contrast to the data found in the group that used the CES‐D scale, with a variation between the lowest and highest AcI with 1.4% to 5.4%^18^, ^19^ respectively (Fig. 2).

It should be noted that the studies classified here in the group ‘diagnostic interviews’ investigated, in addition to the categories of subsyndromal symptomatic depression (SSD) and minor depression (mD), the incidence of major depression (MD).^20^, ^21^, ^22^, ^23^ For this one, a greater variation in AcI between studies can be observed:^22^ with 0.2%,^20^ followed by with 2.5%,^21^ with 5.3% and with 7%.^23^ In terms of comparability with the other groups, the information regarding SSD and mD was used to acquire the AcI values shown in Fig. 2.

As can be seen in Fig. 2, the group of studies that used the GDS to assess the depressive variable can be represented as a group with a greater variation among the AcI values, with 26.6% per year for the study with the highest percentage^16^ and 1.3% per year for the lowest value.^17^ We observed a maximum AcI of 5.4%^18^ for the CES‐D group; 7%^23^ for the group that used diagnostic interviews, and 10.2%^24^ for the group that used other scales.

Regarding the follow‐up time, the group that used the GDS had the lowest mean time, with 2.5 years, followed by the diagnostic interview groups, with 3.8 years; miscellaneous scales, with 5.3 years; and CES‐D, with 6 years. Only two of the included studies carried out a follow‐up of less than a year, respectively 9 and 11 months,^16^, ^25^ which led to an increase in the cI when adjusted by year. These two AcI values were quite different from the value found in the study with the third highest AcI, 12%,^26^ belonging to the GDS group.

As for the time aspect, we observed that 54% of the studies were published between 2016 and 2020,^8^, ^17^, ^18^, ^22^, ^25^, ^26^, ^27^, ^28^, ^29^, ^30^, ^31^, ^32^, ^33^, ^34^, ^35^, ^36^, ^37^, ^38^, ^39^, ^40^, ^41^, ^42^, ^43^, ^44^ 31% between 2011 and 2015,^16^, ^19^, ^20^, ^45^, ^46^, ^47^, ^48^, ^49^, ^50^, ^51^, ^52^, ^53^, ^54^, ^55^ and the minority prior to 2010.^21^, ^23^, ^24^, ^56^, ^57^, ^58^, ^59^ There was no relationship between the time the study was conducted, and the AcI values found.

### Instruments used by the included articles to assess depression

Concerning the instruments used to evaluate the outcome, there was a predominance in the use of scales (91.3%), which function more as a screening tool, but not as a diagnostic one. Among the scales, we observed that the GDS in its several versions (30, 15, and five items) was the most used one (approximately 57%), as well as a change in the cutoff point to define ‘depressive cases’ when using the same version of the instrument.^16^, ^17^, ^26^, ^27^, ^28^, ^30^, ^31^, ^32^, ^33^, ^34^, ^35^, ^37^, ^38^, ^39^, ^40^, ^41^, ^42^, ^45^, ^48^, ^49^, ^50^, ^51^, ^55^, ^57^, ^58^, ^60^

Then, we observed a higher frequency for the CES‐D with 15.2%^18^, ^19^, ^20^, ^43^, ^47^, ^52^, ^53^ and the other scales corresponded to 19.5% of the articles included.^8^, ^24^, ^27^, ^29^, ^36^, ^44^, ^46^, ^56^, ^59^ As for the diagnostic instruments and interviews, they corresponded to 8.3%^20^, ^21^, ^22^, ^23^ of the frequency among the 46 articles included. In the case of the articles that used the CES‐D, heterogeneity was also observed regarding the cutoff point, since some studies used a version with 20 items,^19^, ^47^, ^52^, ^53^ with variable cutoff points including depending on gender, 10 items,^18^ and eight items.^43^, ^61^

Within the articles that used the GDS to assess new cases of older adults with depressive symptoms, 73.1% had an Asian origin,^17^, ^26^, ^30^, ^31^, ^32^, ^33^, ^34^, ^35^, ^37^, ^38^, ^39^, ^41^, ^42^, ^45^, ^49^, ^50^, ^51^, ^55^, ^58^ 15.3% European,^28^, ^40^, ^57^, ^60^ 7.7% came from the American continents^16^, ^48^ and one study from Oceania^25^ that corresponds to 3.9%.

The frequency distribution changes for the other tests were: the CES‐D was more used by European studies, with 71.4%,^19^, ^43^, ^47^, ^52^ followed by China and Taiwan with 28.6%.^18^, ^54^ The group formed from other scales was distributed in descending order to Europe^8^, ^24^, ^56^, ^59^ with 44.5%, Asia^29^, ^44^ and Americas^36^, ^46^ each with approximately 22.2% and one Australian study.^27^

Finally, diagnostic interviews were used by two studies from the United States,^21^, ^23^ South Korea^22^ and France^20^ each at 25%.

### Characteristics of the included studies

The characteristics of each study considered important for this review can be seen in Table 1. We included cohort studies, with follow‐up time varying between 9 months and 16 years, and a variety of nationalities and instruments for outcome assessment.

**Table 1:** Characteristics of the studies included in the systematic review and meta‐analysis

Name of the study, author	Country	Time of follow‐up (years/months)	Exclusion criteria	Sample size (final sample)	Baseline age in years; mean (SD) or %	% women	Outcome; cut point depressive cases	*n* incident cases	Cumulative incidence (cI) %
Aichi Gerontological Evaluation Study (AGES) project; 1. Misawa *et al*. (2019)	Japan	4 Y	Not independently undertaking walking, bathing, or toilet activities in either W1 + 2; mental illness or depression W1, no record for depression questions W2	3464	Young‐old age group (65–74): 74.3%; old‐old (75 or more): 25.7%	47.2	GDS‐15; ≥5	490	14.15
Australian Longitudinal Study of Women's Health (ALSWH); 2. Cations *et al*. (2021)	Australia	15 Y	Not informed	9443	72.6 (1.5)	100	5‐item mental health subscale of the MOS SF 36‐W1; Goldberg Anxiety and Depression Scale	2213	23.43
China Health and Retirement Longitudinal Study (CHARLS); 3. Jing *et al*. (2020)	China	2 Y	Persons aged between 45 and 59 Y	5108	68.02 (6.58)	49.34	CES‐D‐10; ≥ 10	547	10.70
Korean Longitudinal Study on Cognitive Aging and Dementia (KLOSCAD); 4. Oh *et al*. (2020)	South Korea	3.3 Y	NR	3955	70.21 (6.90)	55.5	MINI‐K	358^†^	9.05^†^ 21.70^‡^ (95% CI 19.29–24.12)
Living Profiles of Older People Survey (LPOPS) 5. Roh *et al*. (2015a)	Korea	3 Y	Diagnosed with depression	6647	73.3 (6.2)	55.85	SGDS‐K 15; ≥8	1443	20.75
6. Roh *et al*. (2015a)	Korea	3 Y	No adult children, living with adult children, cognitive imp. at either W, and depression at baseline	4398	69.9 (5.7)	52.6	SGDS‐K 15; ≥8	883	20.1
7. Jin *et al*. (2020)	Korea	3 Y	Visual, hearing, language, cognitive imp. and depression at baseline	6954	69.8 (6.1)	55.5	SGDS‐K 15; ≥8	1504	21.62^§^
National Center for Geriatrics and Gerontology Study of Geriatric Syndromes (NCGG‐SGS); 8. Shimada *et al*. (2020)	Japan	2,5 Y	History of stroke, PD, AD, decline BADL, MMSE scores <18, those certified LTCIS	1792	70.1 (6.3)	46.4	GDS‐15; ≥6	247	13,78 55.1^‡^ (CI 95% 48.7–62.5)
National Free Physical Examination Program in the Hangu Health Center of Tianjin; 9. Chen *et al*.(2019)	China	1 Y	Incomplete date for DS; unable testing: handgrip strength/4‐m walking/measurement of body composition/to communicate with interviewers	691	67.5 (5.7)	56	GDS 30; ≥11	83	12
Obu Study of Health Promotion for the Elderly’ (OSHPE); 10. Makizako *et al*. (2015)	Japan	15 m	History of Parkinson's disease, stroke, depression, Alzheimer's disease, MMSE <18 or depressive symptoms at baseline	3.025	71.4 (5.1)	50,3	GDS 15; ≥6	226	7.47
11. Uemura *et al*. (2018)	Japan	15 m	History of PD, depression, AD, <18 on MMSE, disability (BADL), DS at baseline	3106	71.5 (5.2)	49.1	GDS 15; ≥6	239	7.69
12. Tsutsumimoto *et al*. (2017)	Japan	15 m	Needing support or care by the Japanese Public LTCIS (care level ≥3/5)	3503	71.4 (5.2)	50.3	GDS 15; ≥6	232	6.62
13. Tsutsumimoto *et al*. (2018)	Japan	4 Y	AD, PD, depression; functional disability; need for support or care as result of disability; MMSE ≤18; and DS	2430	71 (4.7)	51.7	GDS 15; ≥6	131	5.39
Progetto Veneto Anziani (Pro.V.A.); 14. De Rui *et al*. (2017)	Italy	4.4 Y	Participants with cognitive imp., depression, severe neurological disease at baseline	891	For groups: improved: 70,9 (3.69); stable: 71.2 (5.2); worsened: 73.5 (6.1)	53.4	GDS 30; ≥11	167	18.74
15. Trevisan *et al*. (2018)	Italy	4.4 Y	Participants with severe cognitive imp.	2104	74.2 (6.8)	62.7	GDS 30; >10	229	10.9
16. Veronese *et al*. (2017b)	Italy	4 Y	Participants with severe cognitive imp.	970	72.5 (6)	54.6	GDS 30; >10	207	21.34^§^
Program 70 y más; 17. Bojorquez‐Chapela *et al*. (2012)	Mexico	11 m	Cognitive imp., GDS ≥6 first W; failed to answer the questionnaires	2661	Group without DS: 69.49; Group with DS: 69.20	Without DS: 44.92; with DS: 58.68	GDS 15; ≥6	645	24.23
Protocolos del Grupo de Investigación em Demencias 10/66; 18. Ortiz *et al*.(2017)	Mexico	3 Y	Depression in the initial evaluation	1512	Groups with incident depression: 65–69 y: 25.1%; 70‐74 y: 32.6%; 75–79 y: 22.5%; 80+ y: 19.8%	65.21	GMS‐AGECAT; ICD 10th edition; DSM‐IV; EURO‐D	227	15.01
Québec Longitudinal Study on Nutrition and Aging (NuAge); 19. Gougeon *et al*. (2014)	Canada	3 Y	Depression or ADMU at baseline, missing/invalid GDS scores or dietary information at baseline or at all three FUA	1358	74 (4)	50.4	GDS 30; ≥11	170	12.51
Survey of Health, Aging and Retirement in Europe (SHARE); 20. Conde‐Sala *et al*. (2019)	13 countries and Israel	2 Y	Not informed	23 201	65–74 years: 46.4; ≥75 years: 3.6%	56.9	EURO‐D 12 items; ≥4	2862	12.33 6.62^‡^ (99.9% CI: 6.61–6.63)
The 3C Study Group; 21. Scali *et al*. (2010)	France	2, 4 Y	Dementia (diagnosed using DSM‐IV revised criteria)	4069 (around 3178 have no DS at baseline)	73.6 (5)	All	CES‐D 20; ≥23	NR	17.4%^§^
22. Godin *et al*. (2012)	France	4, 8, 10 Y	No data on depression, blood pressure, or BMI available. No follow‐up data	4 years‐3090; 8 years‐2359; 10 years‐ 1744	73.5 (4.8)	60	CES‐D Scale 20; 17 point score cutoff for men, 23 for women	478	15.47
23. Carriére *et al*. (2013)	France	Median 9 Y	Prevalent DS or using antidepressants at baseline; visual impairment	2307	65–69: 27.6; 70–74: 35.6; 75–80: 24.5; 80+: 12.3	54.3	MINI; CES‐D 20, ≥16 (severity of DS)	521	22.58
24. Potvin *et al*. (2013)	France	10 Y	Dementia	3390	Groups: no incident depressio*n*: 72.9 (4.7); incident depressio*n*: 73.5 (4.8)	72.5	CES‐D 20; >16 (at least one follow‐up)	491	14.46
The Bambuí Cohort Study of Aging; 25. Nascimento *et al*. (2015)	Brazil	10 Y	Not informed	701	67.4 (6.1)	57.2	GHQ‐12; ≥ 5	256	36.51 4.6%
The English Longitudinal Study of Ageing (The ELSA study); 26. Veronese *et al*. (2017a)	England	2 Y	Depressed at baseline; no data regarding frailty criteria and DS	4077	70.9 (range 60–90)	53	CES‐D 8; ≥4	360	8.83
The Gospel Oak Project VII; 27. Prince *et al*. (1998)	England	1 Y	Not informed	451	75 (8)	61	SHORT‐CARE	46	10.19
The HEIJO‐KYO cohort; 28. Obayashi *et al*. (2018a)	Japan	Median: 23 m	DS or a diagnosis of depression and ADMU at baseline	866	71.5 (6.9)	52	GDS 15; ≥6	75	8.66
29. Obayashi *et al*. (2018b)	Japan	Median: 2 Y	DS and ADMU at baseline	863	71.5 (7)	52.1	GDS 15; ≥6	73	8.45
The JAGES (Japan Gerontological Evaluation Study); 30. Honjo *et al*. (2018)	Japan	Mean: 3 Y (2.6 Y)	Limitations in ADL; with DS at baseline	42 169	Women: 72.7 (5.5), men: 72.6 (5,5)	53.4	GDS 15; ≥5	5257	12.46
31. Tani *et al*. (2016)	Japan	Median: 2.6 Y	Missing responses for both DS or reporting DS at baseline	10 458	65–74: 65.3%; ≥75: 34.7%	43.4	GDS 15; ≥5	1435	13.85^§^
32. Koga *et al*. (2020)	Japan	3 Y	Dependent (ADL) and those receiving Public LTCIB	1737	65–69: 33.2%; 70–74: 30.2%; 75–79: 22.5%; 80–84: 10.8; ≥85: 3.3%	56.6	GDS 15; ≥5	121	6.97
The Kurabuchi Study; 33. Sugai *et al*. (2018)	Japan	2 Y	Hospitalised or institutionalised; GDS≥2 at baseline; insufficient data	548	Age: 65–69: 24.1; 70–74: 31.9; 75–79: 23.9; 80–84: 17.3; ≥85: 7.3	57.11	GDS 5; ≥2 in baseline; GDS 15; ≥6	65	11.86
The Longitudinal Aging Study Amsterdam (LASA); 34. Sanders *et al*. (2012)	Netherlands	16 Y	NR	1928	Total: 68.9 (8.5) Women: 69 (8.3), men 70.6 (8.7)	51.03	CES‐D 20; ≥ 16	469	24
The Niigata Elderly study; 35. Hanindriyo *et al*. (2018)	Japan	3 Y	Lack of data, having a GHQ‐30 >6 or being regular antidepressant users	212	70 years	39,16	GHQ‐30; ≥7	27	12.73
Zhejiang Major Public Health Surveillance Program (ZPHS); 36.	China	1 Y	Dysaudia, cognitive imp. /conditions making difficult to complete the questionnaire/incomplete information; baseline DS	8527	70.4 (7.3)	49.8	PHQ‐9, cutoff score of≥5	732	8.58
Zutphen Elderly Study; 37. Giltay *et al*. (2006)	Netherlands	10 Y^¶^	Missing data on dispositional optimism or risk factors	229	70.8 (4.6)	Not included women	SDS; ≥50	75	32.75
38. Schoevers *et al*. (2000)	Netherlands	3 Y	NR	2244	65–69: 20.6; 70–74: 24; 75–79: 25.9; 80–86: 29.4	62.4	GMS‐AGECAT; ≥3	216	9.62^§^
39. Koizumi *et al*. (2005)	Japan	1 Y	Cognitive Imp (MMSE <18)	475	FU group: 70–74: 51.2; age ≥ 75 years: 48.8	FU group: 49.9	GDS 30; ≥ 11	55	11.6
40. Harris *et al*. (2006)	England	2 Y	Terminal illness or dementia, vision problems, frailty, and living in a nursing home	945	Onset DS: 65–69: 17.03, 70–74: 30.37, 75–79: 24.44, 80–84: 17.14, 85+: 11	41.4	GDS‐15 ≤5 at baseline now, >5 at FU	79	8.36
41. Lyness *et al*. (2009)	USA	4 Y	Current depression at the time of study intake	405 at 1 Y FU; 338 at 2 Y; 259 at 3 Y; 54 at 4 Y	75.6 (6.7)	60 (in 1 Y)	SCID	33	MDD:^‡^, ^§^, ^††^ 5.3
42. Weiss *et al*. (2009)	USA	22 m	NR	512	78.56 (7.22)	76	MINI‐MDE and scored based on DSM‐IV criteria	122	13^‡‡^
43. Chan *et al*. (2012)	China	2 Y	Dementia; bilateral hip replacements or unable to walk independently	2630	71.7 (4.7)	40	GDS 15; ≥ 8	105	4.3^§^
44. Yang *et al*. (2015)	Taiwan	4 Y	DS; dementia or cognitive imp.	1467	Aged 65–74: 49.1; ≥75: 50.9	42.7	CES‐D 10; ≥ 10	215	14.65
45. Yoshida *et al*. (2015)	Japan	3 Y	DS at baseline	680	Groups‐no DS: 72.6 (5.3); DS: 73.3 (5.7)	57.2	GDS 15; ≥6	115	16.9
46. Brown *et al*. (2020)	Australia	9 m	Significant cognitive imp. and patients returning to residential aged care institutions	205	78.38 (7.68)	57	GDS 15; ≥7	37	18.04

Abbreviations: cI, cumulative incidence; CI 95%, confidence interval; NR, not reported; W, wave; MOS SF 36, MOS Short Form 36‐item Health Survey; GDS, Geriatric Depression Scale; CES‐D, Center for Epidemiologic Studies Depression Scale; MINI, Mini International Neuropsychiatric Interview; SSD, subsyndromal symptomatic depression; mDD, minor depressive disorder; MDD, major depressive disorder; PD, Parkinson disease; AD, Alzheimer disease or dementia; MMSE, Mini Mental State Examination; BADL, basic activities of daily living; LTCIS, long‐term care insurance system; DS, depressive symptoms; frailty status: PF, physical frailty; cognitive imp., cognitive impairment; SF, social frailty; GMS‐AGECAT, geriatric mental state‐automatic geriatric examination for computer‐assisted taxonomy; ADMU, antidepressants medication use; FUA, follow‐up assessments; SDS, Zung Self‐Rating Depression Scale; MDE, major depressive episode; GHQ, version of the General Health Questionnaire; BDI, Beck Depression Inventory; SCID, Structured clinical interview for DSM (Diagnostic and Statistical Manual of Mental Disorders); HDS, Hamilton depressive scale; RCI, residential care institution.

^†^ Subsyndromal symptomatic depression + major depressive disorder + minor depression incident cases.

^‡^ Incidence ratio per 1000 people years (CI 95%).

^§^ Value reported in the article, but divergent in the manual calculation: Jin, 2020; Scali, 2011; Tani, 2016; Schoevers, 2000‐elderly with neurotic depressive syndrome; for the purpose of our article, psychotic depressive syndrome was excluded from the calculation; Lyness, 2009‐the sample size at risk was not well described in the paper for each wave; Chan, 2012‐the number of elderly people with depressive symptoms at baseline were excluded from the calculation.

^¶^ Follow‐up time inferred by the authors.

^††^ Only major depressive disorder.

^‡‡^ Major and minor depression.

Of the 46 articles included in this global systematic review, 45 articles investigated community‐dwelling older adults in 13 different countries. A single article included was of a multicentric nature and revealed incidence data of depression/depressive symptoms concerning 14 countries‐13 European and Israel.^8^

**Figure 3:**
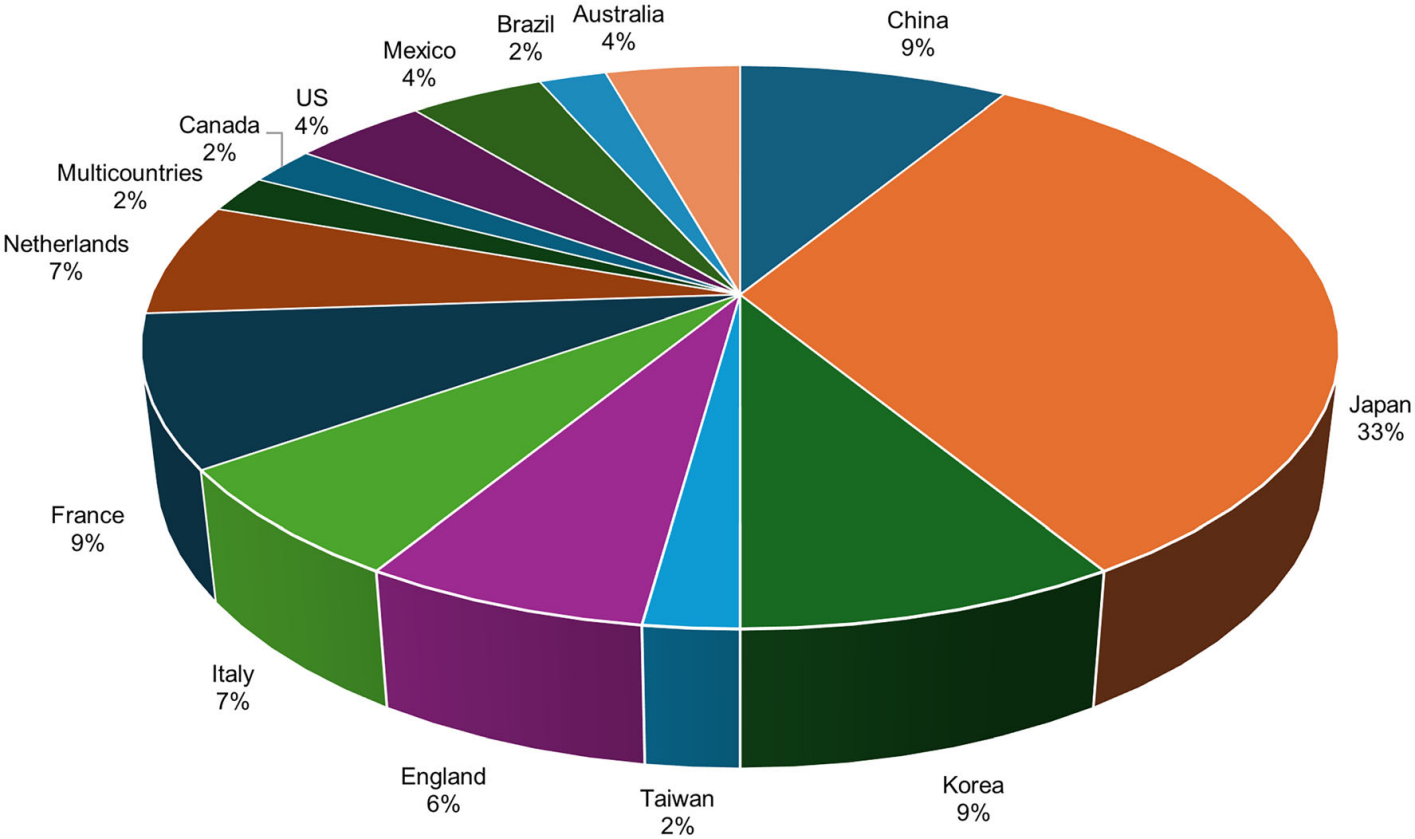
Distribution of publications included in the systematic review by countries.

As shown in the graph (Fig. 3), most of the publications came from the Asian continent (Japan, China, Korea, and Taiwan), with 24 articles included in this systematic review. The second continent to produce more material included in this review was Europe, with a greater distribution of countries: France, England, Italy, the Netherlands, and a multicentre study (the latter included 13 European countries and Israel). Finally, studies with American populations were included in this review (three North American studies, two studies from the continental region of Latin America and the Caribbean) and Oceania (two Australian studies). No studies involving the African continental area or group of countries located on islands were included.

In this systematic review, the group of countries considered developed according to their gross domestic product (GDP) produced 84.8% of the articles.^17^, ^19^, ^20^, ^21^, ^23^, ^24^, ^25^, ^27^, ^29^, ^30^, ^32^, ^33^, ^34^, ^35^, ^37^, ^38^, ^39^, ^40^, ^41^, ^42^, ^43^, ^47^, ^48^, ^49^, ^52^, ^53^, ^55^, ^56^, ^57^, ^58^, ^59^, ^60^

It is noteworthy that about 37% of the included studies explained the exclusion of older adults with severe cognitive impairment or dementia;^16^, ^17^, ^19^, ^25^, ^31^, ^37^, ^40^, ^42^, ^44^, ^45^, ^51^, ^53^, ^54^, ^57^, ^58^, ^60^ 22% of the articles used as exclusion criteria older adults with difficulty or functional disability or who needed definitive financial help from the public security of the country in question.^17^, ^30^, ^32^, ^33^, ^37^, ^38^, ^41^, ^42^, ^45^, ^49^

### Risk of bias among included studies

The assessment of the risk of the studies included in this review can be seen in Table 2. We obtained a score collected from the count of each item fulfilled among the 14, that is, the closer to this total, the fewer biases were observed. We noted that some items were not reported in any of the included articles, such as item 5, in which there should have been mention of issues related to sample calculation or sample power. As for item 12, related to the blinding of interviewers when evaluating the outcome, it was also not observed that the action had been carried out in all the included studies.

**Table 2:** Quality Assessment Tool for Observational Cohort and Cross‐Sectional Studies

Article author, year	1	2	3	4	5	6	7	8	9	10	11	12	13	14	Total
Bojorquez‐Chapela, 2012	Y	Y	Y	Y	NR	Y	Y	Y	Y	N	Y	NR	Y	Y	11
Uemura, 2017	Y	Y	Y	Y	NR	Y	Y	Y	Y	Y	Y	NR	NR	Y	11
Veronese, 2017	Y	Y	Y	U	NR	Y	Y	Y	Y	Y	Y	NR	Y	Y	11
Veronese, 2017	Y	Y	Y	Y	NR	Y	Y	Y	Y	Y	Y	NR	N	Y	11
Prince, 1998	Y	Y	Y	Y	N	Y	Y	Y	N	N	Y	NR	Y	Y	10
Roh, 2014	Y	Y	Y	Y	NR	Y	Y	Y	N	Y	Y	NR	N	Y	10
Roh, 2015	Y	Y	Y	Y	NR	Y	Y	Y	N	N	Y	NR	Y	Y	10
Sanders, 2012	Y	Y	U	Y	NR	Y	Y	Y	Y	N	Y	NR	Y	Y	10
Scali, 2011	Y	Y	Y	Y	N	Y	Y	Y	N	N	Y	NR	Y	Y	10
Schoevers, 2000	Y	Y	Y	Y	NR	Y	Y	Y	Y	N	Y	NR	Y	Y	10
Shimada, 2020	Y	Y	Y	Y	NR	Y	Y	Y	Y	U	Y	NR	N	Y	10
Sosa‐Ortiz, 2017	Y	Y	Y	Y	NR	Y	Y	N	Y	Y	Y	NR	N	Y	10
Sugai, 2018	Y	Y	Y	Y	NR	Y	Y	Y	N	N	Y	NR	Y	Y	10
Tani, 2016	Y	Y	Y	Y	NR	Y	Y	Y	Y	N	Y	NR	N	Y	10
Trevisan, 2017	Y	Y	Y	Y	NR	Y	Y	Y	Y	NA	Y	NR	NA	Y	10
Tsutsumimoto, 2016	Y	Y	NR	Y	NR	Y	Y	Y	Y	N	Y	NR	Y	Y	10
Tsutsumimoto, 2018	Y	Y	Y	Y	NR	Y	Y	Y	N	Y	Y	NR	N	Y	10
Honjo, 2018	Y	Y	Y	N	NR	NR	Y	Y	Y	Y	N	NR	Y	Y	9
Jin, 2020	Y	Y	N	Y	NR	Y	Y	Y	Y	Y	Y	NR	N	N	9
Jing, 2020	Y	Y	Y	Y	NR	Y	Y	Y	N	N	Y	NR	N	Y	9
Koga, 2020	Y	Y	Y	Y	NR	Y	Y	Y	N	N	Y	NR	N	Y	9
Koizumi, 2005	Y	Y	Y	Y	NR	Y	Y	N	Y	N	Y	NR	N	Y	9
Lyness, 2009	Y	Y	N	Y	NR	Y	Y	NR	Y	Y	Y	NR	N	Y	9
Makizako, 2014	Y	Y	Y	Y	NR	Y	Y	N	Y	N	Y	NR	N	Y	9
Misawa, 2018	Y	Y	Y	Y	NR	Y	Y	Y	N	N	Y	NR	N	Y	9
Nascimento, 2015	Y	Y	Y	Y	NR	Y	Y	N	N	N	Y	NR	Y	Y	9
Obayashi, 2017a	Y	Y	Y	Y	NR	Y	Y	N	N	N	Y	NR	Y	Y	9
Obayashi, 2017b	Y	Y	NR	Y	NR	Y	Y	Y	Y	N	Y	NR	N	Y	9
Oh, 2009	Y	Y	Y	Y	NR	Y	Y	Y	N	N	NR	NR	Y	Y	9
Potvin, 2013	Y	Y	NR	Y	NR	Y	Y	Y	Y	N	Y	NR	N	Y	9
Chen, 2018	Y	Y	NR	Y	NR	Y	Y	N	N	N	Y	NR	Y	Y	8
Conde‐Sala, 2019	Y	Y	NR	Y	NR	Y	Y	Y	N	Y	N	NR	N	Y	8
De Rui, 2017	Y	Y	N	Y	NR	Y	Y	N	N	U	Y	NR	N	Y	8
Giltay, 2006	Y	Y	Y	Y	NR	Y	Y	Y	N	N	Y	NR	N	NR	8
Godin, 2012	Y	Y	Y	Y	NR	Y	Y	N	N	N	Y	NR	N	Y	8
Gougeon, 2014	Y	Y	Y	Y	NR	Y	Y	Y	N	N	Y	NR	N	N	8
Hanindriyo, 2018	Y	Y	Y	Y	NR	Y	Y	N	N	Y	Y	NR	N	Y	8
Harris, 2005	T	T	N	Y	NR	Y	Y	Y	N	N	Y	NR	N	Y	8
Carrière, 2013	Y	Y	NR	Y	NR	Y	Y	N	N	N	Y	NR	N	Y	7
Cations, 2020	Y	Y	N	Y	NR	Y	Y	Y	N	N	Y	NR	N	Y	7
Chan, 2012	Y	Y	N	Y	NR	NR	Y	Y	N	N	Y	NR	N	Y	7
Brown, 2020	Y	Y	Y	Y	NR	NR	Y	N	N	N	Y	NR	N	NR	6
Weiss, 2009	Y	Y	U	N	NR	Y	Y	N	N	N	Y	NR	NR	Y	6
Wu, 2019	Y	Y	NR	Y	NR	NR	Y	N	N	N	Y	NR	N	Y	6
Yang, 2015	Y	Y	Y	N	NR	Y	Y	NR	N	N	Y	NR	N	N	6
Yoshida, 2015	Y	Y	N	Y	NR	Y	Y	N	N	N	Y	NR	N	NR	6

Abbreviations: Y, yes; N, no; U, unclear; NA, not applicable; NR, not reported; CD, cannot determine.

## DISCUSSION

This study aimed to synthesise information related to the incidence of depression and depressive symptoms in a specific population of community‐dwelling older adults aged 60 years or over. It is important to highlight that significant heterogeneity characteristics can be observed, especially due to geographical diversity, variation in instruments used, as well as follow‐up time, which makes this systematic review more challenging. However, since many studies met the eligibility criteria and could be included in this review, it was possible to separate the material into groups, which favoured interpretation.

For the evaluation of depression from a dimensional point of view, we found a variety of AcI ranging from 1.3%^17^ to 26.6%,^16^ while in the systematic review by Buchtemann *et al*.,^10^ they observed a variation from 6.1% to 18.7% in incidence proportions. In the categorical assessment of depression, when considering MD, our review observed a variation of 0.2%^22^ to 7.0%,^23^ compared to 1.7% to 7.6% found in the 2012 review.^10^

The difference seen between the ways of defining what is or is not a ‘depressive case’ (whether categorical or dimensional) is not limited to scientific research. It can have repercussions in the demands of the health systems, with a focus on primary care, and in the production of public policies, with a consequent impact on the health of older adults. There is a possibility that the diagnosis of depression in older adults made by diagnostic tools such as International Classification of Diseases^62^ or Diagnostic and Statistical Manual of Mental Disorders^63^ may underestimate cases of depression that are not so severe,^64^, ^65^ but which could benefit from early interventions. Further research would need to be conducted to provide a framework for understanding the early detection of depressive states in older adults.

As for the dimensional depression assessment scales, the GDS in its original version has 30 items and was designed for the specific evaluation of depression in older adults, which made it possible to include more specific items of the population in focus when considering somatic and cognitive complaints, with a sensitivity of 84% and specificity of 95% when considering the cutoff point of 11 or more items.^66^ The 15‐item version was considered in an attempt to include sicker older adults and those with dementia because they have greater difficulty in concentrating for a longer time,^67^ but, as could be seen in the results, it was widely used. It is understood that the diversity of versions and cutoff points present adaptation, validity and reliability studies in each country,^66^, ^67^ but the diversity has been observed as a factor that hinders statistical inferences and comparisons for systematic reviews.

The CES‐D was developed to measure symptomatology in the general population and showed a good level of internal consistency and test–retest stability, including in the older adult population.^68^ Other scales also showed validity for application and especially for assessing the severity of depressive symptoms, such as the Zung Self‐Assessment Scale and the Hamilton Depression Scale.^69^, ^70^

For a few decades, a model of dimensional understanding of depression has been discussed and proposed through follow‐up or trajectory studies of major depressive episodes (MDE). By observing the time in weeks of the depressive level characterised by the severity of symptoms, the authors observed that people diagnosed with MDE spend most of the follow‐up with subsyndromal or minor symptoms when considering a beginning and end of the episode.^71^ This perception reveals a chronic nature of depression, which is associated with low social functioning in adults and older adults.^72^ Furthermore, it has been observed in older adults that even moderate depressive symptoms lead to losses related to years lived with quality.^3^

The dimensional grasp allows the understanding of the depressive construct as a spectrum, which can locate an individual according to the symptomatologic severity in depressive symptoms, minor depression, dysthymia, and major depressive disorder.^73^, ^74^ Therefore, the assessment of the depressive construct through the means of validated scales can be an accessible tool for primary care services,^73^ especially to target older adults who are at higher risk.^68^

A different profile of included articles was observed in another systematic review conducted^10^ when compared to ours. In Buchtemann *et al*.'s review, which also sought to assess the incidence of depression and depressive symptoms in community‐dwelling older adults, it was observed that 75% of the included materials dealt with data from European countries, with the rest consisting of North American studies and a minority from Asia or Oceania. This totality of articles conducted in countries considered developed differed from ours, as it was possible to include studies carried out in developing countries, in addition to the axis of the group of developed countries being predominant in the Asian continental area.

Moreover, there is a difference in the number of articles included in the present review and in that of Buchtemann et al.^10^ regarding the way of researching the depressive variable: in our study, most of the articles included used dimensional assessment, obtained through the application of scales, contrary to what was observed in the 2012 review, with most of the material included consisting of categorical diagnosis.

Concerning geographic influence, the United Nations classification was chosen regarding continental regions: Africa, Asia, Latin America and the Caribbean, North America, and Oceania.^75^ In this review, we observed a considerable asymmetry when comparing the amount of published material between the countries considered developed and less developed. This difference may be related to the timing and speed of population growth in certain geographic areas. For example, sub‐Saharan African countries are at a moment of accelerated growth, and do not have the same proportional frequency of older adults compared to those countries that have already been going through an ageing process for a few decades.^1^ However, this does not seem to be the only justification. Countries that are part of the lowest GDP group had participation in this review (or did not participate at all) and represented a large amount of the global population in 2019, such as China, India, and Brazil.^1^

In addition, it can be observed that the Asian continent collaborated with a large part of the articles included, with emphasis on materials from Japan. It is known that Japan and the administrative regions of China such as Hong Kong and Macau are the places with the highest life expectancy at birth in the world.^1^

In this review, most of the articles came from countries considered developed by the United Nations Organizations.^75^ In recent years, these countries have already presented a large proportion of older adults in their populations. According to information from the Organization for Economic Cooperation and Development,^76^ among the developed countries participating in this systematic review, the highest proportion of older adults by 2021 was observed in Japan, with 28.9% of the population composed of older adults aged 65 years or older. Italy ranks second in terms of the proportion of older adults with 23.7%, followed by other European countries, which include France, the Netherlands, and England with 20.9%, 19.9%, and 18.8% respectively. On the American continent, Canada and the United States have a proportion of older adults of 18.5% and 16.5% of the population, and in Oceania, Australia had a proportion of 16.8% in 2021. Korea and Taiwan (https://www.statista.com/) had 16.6% and 16.8% respectively of older adults in their territories.

It is possible to hypothesise that there is a greater interest in fostering scientific research in developed countries, which enables large studies such as those observed in this systematic review. The evaluation of the occurrence of DDS on a global level and the geographic extension obtained can be considered a strong point in this systematic review of incidence.

From the standpoint of the quality of the included studies, most were characterised by good internal validity, which allows confidence in the exposed results at the end of the reading. It can be observed that for item 5, in which it was sought how these articles reached the sample size, the sample power, and the estimated effect size, almost none of the studies scored, which may be explained by the exploratory nature of the studies. Item 12, which asked about the blinding of the evaluators in the data collection process, was not covered by any of the included studies. It is possible that the description was not made because it is inferred that there is already a kind of blinding in cohort studies, especially in research with large populations, in which the outcome evaluator does not know the status at baseline.

Still regarding bias assessment, among the studies that obtained total scores of 6 or less, we could observe that flaws or gaps in the description and transparency may have influenced this result, and in the end, the admission by the evaluators that the data revealed were in fact true was impaired. It is emphasised that future cohort studies should be careful to make it as clear as possible that the exposure comes before the outcome in a similar sample population.

It is worth pointing out that there was a variation in the follow‐up period between the studies, which did not make it feasible to estimate the DDS per group through meta‐analysis, which can be highlighted as an important limitation of this manuscript. Another limitation is the variety of instruments that assess the depressive attribute, which generated heterogeneity. Despite this, the grouping by instruments and the annual adjustment proposed for the cI allowed some comparability among the studies. An additional constraint was the exclusion of the grey literature due to a large number of articles already included in the previous steps, which may be associated with a selection bias. Furthermore, due to the number of articles included, it was not feasible to evaluate the lists of articles included to check eligibility.

Finally, as this is a review at a global level, a large volume of studies about depression in community‐dwelling older adults in the last decades was found. This amount of production occurred predominantly in countries considered developed from both social and economic perspectives, and whose demographic transition is already a reality. This study has shown that the global AcI of depression and depressive symptoms occurs at around 3.9% to 4.5%, which in turn is associated with the impairment to older adults' general health. This reinforces the need for health policies for older adults to also incorporate mental health care, especially for preventive and curative actions for depression or clinically significant depressive symptoms.

## AUTHOR CONTRIBUTIONS

**L. E. E. Brasileiro:** conceptualisation; data curation; formal analysis; investigation; methodology; project administration; resources; supervision; validation; visualisation; writing – original draft; writing – review and editing. **Amanda Almeida Gomes Dantas**: investigation; supervision; validation; visualisation; writing – review and editing. **Dorothy Bezerra Linhares:** investigation; validation; visualisation. **Heron Alves Vale:** investigation; validation; visualisation. **Marc Terradas‐Monllor:** investigation; validation; visualisation; writing – review and editing. **Mirari Ochandorena‐Acha:** investigation; validation; visualisation; writing – review and editing. **Aílla Lorenna de Medeiros Paiva:** investigation; validation; visualisation. **Maria Yasmin Dantas de Medeiros:** investigation; validation; visualisation. **Javier Jerez‐Roig:** investigation; methodology; project administration; resources; supervision; validation; visualisation; writing – review and editing. **Dyego Leandro Bezerra de Souza:** conceptualisation; funding acquisition; investigation; methodology; project administration; resources; supervision; validation; visualisation; writing – review and editing.

## DISCLOSURE

The authors do not have any conflicts of interest to disclosure.

## FUNDING INFORMATION

Dyego Leandro Bezerra de Souza thank CNPq (Brazilian National Council for Scientific and Technological Development) pro‐ ductivity grants 308168/2020‐8.

## Data Availability

The data that support the findings of this study are available on request from the corresponding author. The data are not publicly available due to privacy or ethical restrictions.
